# Evaluation of clinical and serological responses after full-mouth implantation in single-visit versus multiple-session surgery

**DOI:** 10.34172/japid.2024.005

**Published:** 2024-03-24

**Authors:** Atabak Kashefimehr, Amirreza Babaloo, Ahmad Afrashteh

**Affiliations:** Department of Periodontics, Faculty of Dentistry, Tabriz University of Medical Sciences, Tabriz, Iran

**Keywords:** Dental implant, Inflammatory markers, Full-mouth implant, Wound healing

## Abstract

**Background.:**

This research aimed to evaluate the clinical characteristics of pain and wound healing and serological inflammatory markers after full-mouth implantation in one session compared to several sessions.

**Methods.:**

A single-masked clinical trial was conducted on 20 patients (n=10) receiving full-mouth implants. Patients were randomly divided into two groups. The first group was operated under general anesthesia in one session and the second group in multi-sessions. Inflammation level was evaluated through white blood cell (WBC) and serum C-reactive protein (CRP) before and after surgery by a blood test. Pain and early wound healing (EHS) assessment was conducted after surgery with VAS and EHS indicators, respectively. Serological and clinical parameters were compared by repeated-measures ANOVA and Sidak and Man-Whitney U tests, respectively, using SPSS 20.

**Results.:**

The CRP level 48 hours postoperatively was not different in the two groups; however, seven days after treatment, it was higher in the multi-session group than in the single-session approach. The WBC was not different between the two groups at evaluated intervals. Serum levels of WBC and CRP increased 48 hours postoperatively and decreased seven days later. EHS showed no difference between the two groups at the three investigated intervals. The amount of VAS 24 and 48 hours and 7 days postoperatively was higher in multi-session surgery than in the one-session approach. In both groups, VAS was not different at 24 and 48 hours postoperatively and decreased over seven days.

**Conclusion.:**

Full-mouth implant surgery under general anesthesia in one session caused less inflammation and pain postoperatively while presenting the same wound-healing process as the multi-session surgery

## Introduction

 Patient demand for one-session dental implant surgery with fewer complications is increasing. Placing implants in one session reduces the chair side time and the possibility of infection. It effectively decreases the trauma and stress to the patient compared to surgery in several sessions. Endosseous implants’ healing involves hard and soft tissues, proceeding through inflammatory, proliferative, and remodeling phases. The inflammatory phase initiates wound healing right after the first injury through hemostasis, coagulation, and increased chemotaxis, which causes symptoms like pain, swelling, or redness.^1^

 Clinicians desire to accurately prognosticate the treatment outcomes and develop predictable dental implant surgical protocols. Long- and short-term postoperative healing outcomes are unpredictable; however, clinical studies can evaluate different phases of early wound healing (EHS).^2-4^ Evidence-based studies contribute to assessing various treatment modalities to pursue the foremost approach with fewer complications. Different serum, salivary, and gingival crevicular fluid biomarkers have been evaluated to assess the presence and intensity of inflammation in the oral surgical area.^5,6^ Among these, interleukin-1β, interleukin-6, tumor necrosis factor-α, C-reactive protein (CRP), and white blood cells can be mentioned.^5,7^ According to previous studies, inflammatory biomarker levels in saliva and serum change due to chronic periodontitis or peri-implantitis.^8^ Moreover, Chaushu et al^9^ demonstrated a strong correlation between blood cell counts and peri-implantitis. Similar to all the mentioned conditions, implant surgery, flap design, and insertion protocol affect the systematic inflammation, evaluated by biomarkers and leukocyte count, and consequently, the soft tissue healing and remodeling procedure.^10^

 Full-mouth implant placement includes edentulous patients or those with few teeth and poor prognoses and extraction treatment plans. Considering the number of implants placed in these patients, the amount of stress and trauma inflicted on the patient is very significant. These factors affect pain, inflammation, and wound healing in the surgical area. In the present study, the inflammation biomarker CRP and the number of white blood cells were used to evaluate the level of inflammation following implant surgery. Furthermore, the pain inflicted on the patient and the wound healing of peri-implant tissues were assessed after full-mouth surgery conducted in one or multiple sessions.

## Methods

###  Study design and patient selection

 Briefly, 20 edentulous subjects who had accepted a treatment plan for full-mouth implant placement and were referred to the Periodontics Department, Faculty of Dentistry, Tabriz University of Medical Sciences between March 2023 and September 2023 were included.

###  Inclusion criteria

- Patients in need of 12‒14 implants
- Patients aged ≥ 18 years
- Systemically healthy patients
- Patients who were periodontally healthy or had mild gingivitis
- Patients with plaque index or bleeding of probing less than 20%
- Patients able and willing to provide informed consent

###  Exclusion Criteria

- Patients taking antibiotics for the last six months
- Those who had allergies to amoxicillin or other medications
- Patients with any systemic conditions affecting the healing process
- Smokers
- Patients in need of simultaneous hard or soft tissue grafting
- Patients unable or unwilling to comply with study procedures and visits

###  Implant surgery protocol

 The patients were randomly divided into two groups. In group A (10 samples), the surgical procedure was performed under general anesthesia. The full-mouth implants (DIO Implant, Korea) were placed all in one session. In edentulous areas, access was gained by crestal incision followed by flap elevation. The extraction of hopeless teeth/roots and immediate implantation were performed during the same session.

 In group B (10 samples), the surgical procedure was performed under local anesthesia. The implants (DIO Implant, Korea) were placed in several sessions at one-week intervals. Generally, implants of each quadrant were placed in one session. Access was gained by crestal incision followed by flap elevation. After implant placement, the mucosa was adapted to cover the graft and sutured with a 3-0 black silk suture.

 Four basic principles were assured during the surgery:

1. Canine teeth and first molars were considered key positions.
2. Absence of more than three adjacent pontics.
3. Reduction and preferably elimination of cantilevers.
4. At least one implant was placed in each segment of the dental arch.

Patients in both groups were then advised to follow standard postoperative instructions.

###  Early wound healing evaluation

**Figure 1:**
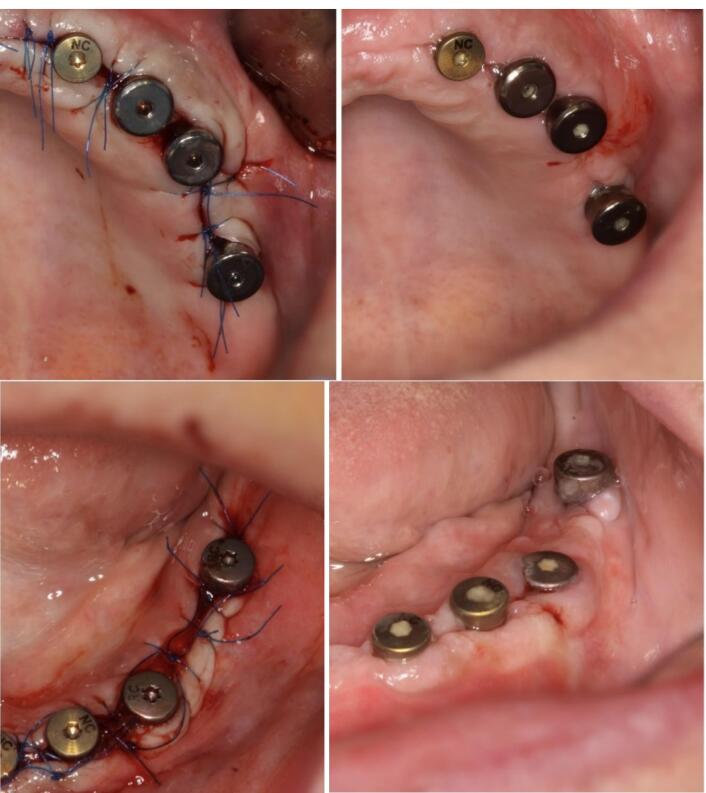
Full-mouth implant placement immediately after surgery (right) and one week after surgery (left)

 Patients were recalled one week after surgery (Figure 1), and a trained clinician evaluated early wound healing using the EHS scores. The EHS is composed of three parameters: clinical signs of re-epithelization (CSR), clinical signs of hemostasis (CSH), and clinical signs of inflammation (CSI).^1^

###  Pain assessment

 Subjects were trained to select a whole number ranging between 0 and 10 that best represented the intensity of their perceived pain, with 0 and 10 representing no and worst possible pain, respectively. Pain assessment was done 24 and 48 hours and 1 week after surgery.

###  Peripheral blood sampling

 Peripheral blood samples were obtained 24 hours before and 48 hours and 1 week after surgery by a second professional clinician. Blood was drawn into a 5-mL vial using a blood collection set and immediately sent to the laboratory at 4 °C. The serum was separated the next day and kept frozen at −20 °C until testing.

###  ELISA

 Commercially available quantitative sandwich enzyme-linked immunoassay kits were used to detect CRP levels in serum samples, according to the manufacturer’s instructions. Each sample was tested three times to reduce the error.

###  Statistical analysis

 Serological data were compared between the two groups using independent samples t-test, repeated-measures ANOVA, and post hoc Sidak test. Clinical indicators were compared by the Man-Whitney U test using SPSS 20.

## Results

 The mean age of the enrolled participants in the single- and multi-session groups was 55.88 ± 5.84 and 59.80 ± 7.15, respectively, with no significant difference between them (*P* = 0.228).

 The Kolmogorov-Smirnov test was used to check the normality of the data distribution.The results showed that the distribution of variables under investigation at all the time intervals was normal (*P* > 0.05). Therefore, parametric tests were used for comparison.

###  Serological evaluation

 The concentrations of CRP and *white blood cell* (WBC) at three time intervals are presented in Table 1. According to the repeated-measures ANOVA, there was a significant difference in the mean CRP values between the two groups, with lower values in the one-session group than in the multi-session group (*P* < 0.05). Repeated-measures ANOVA and post hoc Sidak test showed that the mean CRP and WBC levels 24 hours after surgery were significantly lower than after 48 h and 1 week (*P* < 0.001), with higher values 48 hours after surgery than after 7 days (*P* < 0.001).

**Table 1:** Means and standard deviations of CRP and WBC levels at the three time intervals

**Evaluation time**	**Group**	**CRP**			**WBC**		
		***P *** **value**	**SD**	**Mean**	***P*** ** value**	**SD**	**Mean**
24 hours before surgery	One-stage	0.001	0.82	5.70	0.410	1.04	6.93
	Multi-stage		2.64	9.50		1.03	7.32
	Total		2.72	7.60		1.02	7.13
48 hours after surgery	One-stage	0.573	4.76	19.00	0.547	1.05	7.46
	Multi-stage		4.59	20.20		1.06	7.75
	Total		4.59	19.60		1.04	7.61
One week after surgery	One-stage	0 > .001	2.27	8.50	0.514	0.94	7.22
	Multi-stage		3.55	14.20		1.07	7.52
	Total		4.12	11.35		0.99	7.37
Total	One-stage		6.54	11.07		1.00	7.20
	Multi-stage		5.70	14.63		1.03	7.53

 According to the independent samples t-test, the mean CRP variable in the one-stage group was significantly lower than in the multi-stage group 24 hours and 7 days after surgery (*P* < 0.05); however, there was no significant difference between the mean CRP variable between the two groups 48 hours after surgery (*P* > 0.05). The WBC count was not significantly different between the two groups in all three time intervals (*P* < 0.001).

###  Clinical evaluation

 The results of EHS scores are presented in Table 2. Mann-Whitney U test results showed no significant differences in CSR, CSH, and CSI scores between the two groups (*P* > 0.05).

**Table 2:** EHS parameters in one-session and multi-session full-mouth implant surgeries

		**Sum of points**	**Mean points**	**n**	**Mann-Whitney U test**	**Z**	***P*** ** value**
Clinical signs of reepithelization	One-stage	105.00	10.50	10	50.00	0.000	1.000
	Multi-stage	105.00	10.50	10			
	Total			20			
Clinical signs of hemostasis	One-stage	120.00	12.00	10	35.00	-1.371	0.170
	Multi-stage	90.00	9.00	10			
	Total			20			
Clinical signs of inflammation	One-stage	122.00	12.20	10	33.00	-1.594	0.111
	Multi-stage	88.00	8.80	10			
	Total		10.50	20			

 The results of the Mann-Whitney U test (Table 3) showed that in all three evaluation intervals, there was a significant difference between the average rating of the VAS variable between the two groups, and the average rating of the VAS in the one-stage group was lower than that in the multi-stage group (*P* < 0.05).

**Table 3:** VAS rating in one-session and multi-session full-mouth implant surgeries

		**n**	**Mean points**	**Mann-Whitney U test**	**Z**	***P*** ** value**
24 hours after surgery	One-stage	10	7.55	0.021	-2.301	20.50
	Multi-stage	10	13.45			
	Total	20				
48 hours after surgery	One-stage	10	7.60	0.021	-2.317	21.00
	Multi-stage	10	13.40			
	Total	20				
One week after surgery	One-stage	10	8.00	0.042	-2.038	25.00
	Multi-stage	10	13.00			
	Total	20				

 In each of the two groups, Friedman’s test showed a statistically significant difference between the average ratings of the VAS variable at the three assessment intervals (*P* < 0.05). Further assessment by Wilcoxon test showed no statistically significant difference between the average rating of the VAS 24 and 48 hours after surgery (*P* > 0.05); however, the average VAS rating 24 and 48 hours after surgery was significantly higher than the rating 7 days after surgery (*P* < 0.05).

## Discussion

 It is crucial to control inflammation after dental implant surgery to reduce bone loss, maintain the health of the surrounding soft tissue, and increase the efficiency and survival of implants. It has always been a question whether placing all implants in full-mouth surgery cases should be done in multiple sessions or whether it is advantageous to do the surgery in one session. The scope of full-mouth implant surgery in one session is large, and the inflammation level in the serum, saliva, and soft and hard tissues around the implant may increase due to the high level of injury. Moreover, the amount of pain suffered by the patient leaves room for questions when choosing between one-session or multi-session implants. Notwithstanding, fewer surgery sessions mean there is no need to repeat the stress on the patient and reduce the cost and patient’s chair side time. In addition, increasing the number of sessions is associated with changes in crestal bone characteristics or regional bone loss.^11^ Since no research has been done so far to compare the serological and clinical features between single and multi-session full-mouth implant surgeries, the purpose of this research was to evaluate the clinical features of pain, wound healing, and serological inflammation in full oral implant surgery in one session compared to several sessions.

 Extracting a tooth or creating a flap for surgery starts a series of inflammatory processes, epithelization, and remodeling of bone and soft tissue. Previous studies have shown no difference between bone and soft tissue characteristics following implant placement immediately after tooth extraction or four months later. Therefore, immediate implant placement reduces the number of surgeries by reducing pain and the number of patient visits.^12,13^ CRP and WBC serum markers were used to investigate the body’s inflammatory response to implant and third molar surgery and chronic periodontitis.^14-16^ The present study showed that the serum level of CRP was generally higher in the group that had multiple sessions for full-mouth implant surgery. A more detailed examination of this finding showed that the level of CRP 48 hours after surgery was not different in the two groups. However, seven days after surgery, it was higher in the multi-session group than in the single-session group. This is clearly due to the increase in the number of sessions and repetition of the wound and repair process in the surgical area. The results of the present study showed that 48 hours after surgery in both groups, the CRP level increased significantly and decreased for the next seven days; however, it was still significantly higher than the preoperative evaluation. Previous studies have often investigated the serum CRP levels in patients with periodontitis or peri-implantitis, reporting higher levels of this marker in the serum of patients with inflamed gingival tissue or periodontal ligament.^5,7^ This shows that the inflammation of the soft tissues in the oral cavity increases the CRP marker in the serum. Ur Rahman et al^6^ showed that CRP levels in the serum of patients with periodontitis were higher than normal. However, after the extraction of teeth with poor prognosis and implantation of implants, the serum CRP level decreased. This marker decreased significantly during 12 months of follow-up after implant surgery. Moreover, Singh et al^15^ showed that the serum CRP levels increased after the third molar removal surgery, indicating its role in the inflammatory response. Next, the inflammatory response was evaluated by WBC counts. Studies have shown that the level of WBC is directly related to the level of serum inflammation and infection.^17^ In the current study, the serum levels of WBC at all three time intervals (before, 48 hours, and 7 days after surgery) were not significantly different between single and multi-session surgery groups. However, in both groups, the WBC level was higher 48 hours after surgery than before surgery, and this value decreased up to seven days later. Therefore, it can be noted that the level of serum inflammation and infection is low, provided the sterilization principles do not affect the WBC count during one or more surgery sessions. Chaushu et al^9^ showed that the WBC levels increased after peri-implantitis and decreased after debridement and treatment.

 As an important evaluation method for choosing the implant surgery protocol, examining wound healing indices showed no difference between the three clinical indices of re-epithelialization, inflammation, and hemostasis between single- and multi-session surgery groups. Studies have shown that establishing the biological width and improving the soft tissue around the implant takes up to 6‒8 weeks.^18^ The inflammatory phase starts parallel to the homeostasis and continues throughout the surgery. This phase, which starts with the release of chemokines and the recruitment of neutrophils, is necessary for the next phase, which is the epithelium regeneration. The last phase in wound healing is long-term remodeling, which might take months.^3^ One of the reasons why the wound healing indices in the current study were the same between single- and multi-session surgery is that the oral tissue can regenerate more and faster than other tissues, such as the skin, and the amount of formed scar tissue is much less.^19^ It should be noted that various factors affect the healing process, including the conditions of creating a flap or flapless surgery, the degree of soft tissue destruction, and even the topography of the used implant. For instance, it has been shown that wound healing and vascularization occur faster and more frequently in flapless and one-stage surgeries.^20^ The high CRP levels in the multi-session group, as shown previously, indicated that the inflammation might be associated with the bone healing process or frequency of surgery and flap insertion. Mueller et al^21^ showed that flapless implant surgery is associated with faster wound healing, better hemostasis, and less inflammation. This study confirmed that the flapless group had better re-epithelialization 1, 2, 4, and 12 weeks after surgery.

 The present study showed that the pain suffered by the patient (VAS index) in the three investigated time intervals, 24 hours, 48 hours, and 7 days after surgery, was significantly higher in multi-session surgery than in the single session. As reported, the rate of wound healing was the same in both groups; therefore, the pain endured by the patient was due to the increase in the number of surgical sessions and the increase in stress and anxiety of the patient. Furthermore, the operation under general anesthesia reduces the patient’s stress and anxiety.^22^ It should be mentioned that in both types of surgery, the maximum amount of pain was recorded 24 and 48 hours after surgery, which is due to the applied flap and the pressure resulting from placing the implant in the bone. The severity of pain in these two periods was the same between the two groups, and after that, it decreased within seven days with the acceleration of wound healing. Da Cunha et al^23^ reported that patients with full-mouth implant treatment plans should expect pain after surgery. However, Gómez-de Diego et al^24^ showed that the amount of stress and anxiety before surgery is related to the amount of pain after it, and for this reason, increasing the number of surgical sessions, despite the lack of significant change in the wound healing process, increases the amount of pain inflicted on the patient. Hashem et al,^25^ in line with the present study, showed that if there are no signs of inflammation and infection, the pain decreases significantly within six days after implant surgery. González-Santana et al^26^ investigated the relationship between the amount of pain and swelling following implant surgery and the number of implants. The results indicated that the pain reported by the patient was directly related to the number of implant surgeries. These results are consistent with the present study. Also, similar to the present study, it was reported that the level of inflammation was the highest possible 48 hours after surgery and gradually decreased after that. They reported that patients receiving full-mouth implants felt more pain than those receiving single-unit implants.A meta-analysis study by Gao et al^27^ showed that the patient’s pain level is directly related to the extent of the surgery. Flapless surgery with a smaller surgical extent causes less pain to the patient one day after the surgery; however, contrary to the present study, the patient’s pain during the next three days was not different between the two groups. Notwithstanding, Pal et al^28^ reported that patients’ pain level following immediate implant placement after the tooth extraction was less than that of the group in which the implants were placed during several sessions. In this study, the difference in pain continued for one week, after which there was no significant difference in pain between the two groups, which was attributed to wound healing. Also, the amount of swelling, inflammation, and marginal bone loss in the second group was higher than in the first group.

 As can be seen from the results, the amount of serum inflammatory marker (CRP) and patient’s perceived pain in the group that received full-mouth implant during several sessions was higher than the patients who underwent surgery during one session. Since the index of wound healing was the same in both groups, surgery for patients without teeth or patients who receive more than one implant unit is recommended during one session rather than several sessions. For future studies, it is recommended that other inflammatory markers and clinical markers be evaluated at each interval in the multi-session surgery group in order to investigate the progress of the level of markers during treatment.

## Conclusion

 The amount of serum inflammatory marker (CRP) and patient’s pain in full-mouth implant surgery was higher when implants were placed during several sessions than in one session. Since the wound healing process was not different in the two groups, full-mouth implant surgery of edentulous patients during one session is suggested due to less inflammation and pain.

## Competing Interests

 The authors declare no competing interests.

## Data Availability Statement

 The datasets used and/or analyzed during the current study are available from the corresponding author upon reasonable request.

## Ethical Approval

 The study protocol and informed consent forms were approved by the Research Ethics Committee of Tabriz University of Medical Sciences (approval no: IR.TBZMED.REC.1401.713). All the participants provided written informed consent forms before being included in the study.

## Funding

 This study was supported by Tabriz University of Medical Studies.
